# Açaí (*Euterpe oleracea* Mart.) Modulates Oxidative Stress Resistance in *Caenorhabditis elegans* by Direct and Indirect Mechanisms

**DOI:** 10.1371/journal.pone.0089933

**Published:** 2014-03-03

**Authors:** Larissa de Freitas Bonomo, David Nunes Silva, Patrícia Ferreira Boasquivis, Franciny Aparecida Paiva, Joyce Ferreira da Costa Guerra, Talita Alves Faria Martins, Álvaro Gustavo de Jesus Torres, Igor Thadeu Borges Raposo de Paula, Washington Luiz Caneschi, Philippe Jacolot, Nicolas Grossin, Frederic J. Tessier, Eric Boulanger, Marcelo Eustáquio Silva, Maria Lúcia Pedrosa, Riva de Paula Oliveira

**Affiliations:** 1 Núcleo de Pesquisa em Ciências Biológicas, Universidade Federal de Ouro Preto, Ouro Preto, Brazil; 2 Departamento de Ciências Biológicas, Universidade Federal de Ouro Preto, Ouro Preto, Brazil; 3 Departamento de Biodiversidade, Evolução e Meio Ambiente, Universidade Federal de Ouro Preto, Ouro Preto, Brazil; 4 Institut polytechnique LaSalle Beauvais, Beauvais, France; 5 Faculté de Médecine – Pôle Recherche, Université de Lille 2, Lille, France; 6 Departamento de Alimentos, Universidade Federal de Ouro Preto, Ouro Preto, Brazil; CSIR-Central Drug Research Institute, India

## Abstract

Açaí (*Euterpe oleracea* Mart.) has recently emerged as a promising source of natural antioxidants. Despite its claimed pharmacological and nutraceutical value, studies regarding the effects of açaí *in vivo* are limited. In this study, we use the *Caenorhabditis elegans* model to evaluate the *in vivo* antioxidant properties of açaí on an organismal level and to examine its mechanism of action. Supplementation with açaí aqueous extract (AAE) increased both oxidative and osmotic stress resistance independently of any effect on reproduction and development. AAE suppressed bacterial growth, but this antimicrobial property did not influence stress resistance. AAE-increased stress resistance was correlated with reduced ROS production, the prevention of sulfhydryl (SH) level reduction and *gcs-1* activation under oxidative stress conditions. Our mechanistic studies indicated that AAE promotes oxidative stress resistance by acting through DAF-16 and the osmotic stress response pathway OSR-1/UNC-43/SEK-1. Finally, AAE increased polyglutamine protein aggregation and decreased proteasome activity. Our findings suggest that natural compounds available in AAE can improve the antioxidant status of a whole organism under certain conditions by direct and indirect mechanisms.

## Introduction

Açaí (*Euterpe oleracea* Mart.) is an exotic fruit originally native to Central and South America that grows in the floodplains of the Amazon region [1]. Traditionally, açaí is used as a medicinal plant and as a staple food in many parts of Brazil. In recent years, açaí pulp has gained international attention as a functional food due to its nutritional benefits and therapeutic promise. Composition analysis shows that açaí pulp contains approximately 13% protein, 48% lipids, 1.5% total sugar and several other nutrients such as lignans, dietary fiber and polyphenols [2]. The main polyphenols found in açaí are anthocyanins, proanthocyanidins (specifically cyanidin 3-O-glucoside and cyanidin 3-O-rutinoside) and other flavonoids [2], [3]. The overall pharmacological properties of açaí are related to its antiproliferative, anti-inflammatory, antioxidant and cardioprotective effects [4].

Açaí pulp and its polyphenolic fractions show high antioxidant activity based on various *in vitro* assays, predominantly against DPPH, superoxide and hydroxyl radicals and hypochlorous acid [5]–[11]. *In vitro* cell-based assays have also demonstrated that the pulp can reduce ROS production in human erythrocytes and polymorphonuclear (PMN) cells exposed to oxidative stress [8], [9]. Brain tissue cells pretreated with açaí decreased H_2_O_2_-induced damage to lipids and proteins and reduced the activities of the antioxidant enzymes superoxide dismutase (SOD) and catalase (CAT) to basal levels [12].

Despite its claimed pharmacological and nutraceutical value, studies regarding the effects of açaí *in vivo* are limited [4]. Dietary açaí in rats reduces ROS production by neutrophils, increases GSH levels, reduces protein damage and activates the gene expression of glutathione peroxidase (GPx) and γ-glutamylcysteine synthetase (γ-GCS) in the liver [13]. In humans, intervention studies of volunteers ingesting an açaí juice blend showed a significant increase in serum antioxidant capacity and a reduction of lipid peroxidation [8], [14]. In another study, açaí puree improved select markers of metabolic disease risk in overweight adults [15]. The high superoxide and peroxyl radical scavenging capacity of açaí pulp suggests that açaí has anti-aging properties. In *Drosophila melanogaster*, dietary açaí supplementation increased the lifespan of female flies fed a high fat diet and also under oxidative stress induced by *sod1* RNAi [16]. In another fruit fly, *Anastrepha ludens*, açaí supplementation promoted survival in flies on diets with high fat and high sugar while decreasing lifetime reproductive output [17].

In addition to flies, genetic and pharmacological modifications of stress resistance and lifespan mechanisms have been well elucidated in the nematode *Caenorhabditis elegans*. A number of genes and pathways have been identified to modulate lifespan in *C. elegans*. These highly conserved pathways include the insulin/IGF-1 receptor-like signaling pathway, which depends on the transcription factor DAF-16/FOXO. Reduced insulin/IGF-1 signaling activates DAF-16 nuclear localization, which in turn induces the expression of genes that increase lifespan and promote resistance to various stresses [18]. SKN-1/Nrf is another transcription factor regulated by the insulin/IGF-1 signaling pathway [19]. SKN-1 contributes to the increased stress tolerance and longevity resulting from reduced insulin/IGF-1 signaling independently of DAF-16 [19]. Using this model, several studies have demonstrated the beneficial effects of natural products in promoting stress resistance and longevity, including extracts from *Ginkgo biloba* [20], blueberry [21], *Cinnamomum cassia* bark [22] and cranberry [23].

In the present study, we use the *C. elegans* model to evaluate the *in vivo* antioxidant properties of açaí on an organismal level and to unveil its mechanism of action. Our results indicate that açaí aqueous extract (AAE) increases both oxidative and osmotic stress resistance independently of any effect on reproduction and development. AAE also suppressed bacterial growth, but this antimicrobial property did not influence stress resistance. AAE-increased stress resistance was correlated with reduced ROS production, the prevention of SH level reduction and *gcs-1* activation under oxidative stress conditions. AAE promotes oxidative stress resistance by acting through DAF-16 and the osmotic stress response pathway OSR-1/UNC-43/SEK-1. Our findings suggest that AAE modulates oxidative stress responses *in vivo* by both direct and indirect mechanisms.

## Materials and Methods

### Chemicals and Reagents

M199, Fungizone, penicillin, streptomycin and L-glutamine were purchased from Gibco by Life Technologies (Saint Aubin, France). Methanol, HPLC-grade acetonitrile, HPLC-grade water, formic acid, DPPH (2,2-diphenyl-1-picryl-hydrazyl), Trolox (6-hydroxy-2,5,7,8-tetramethylchroman-2-carboxylic acid) and cyanidin 3-O-rutinoside and kanamycin were purchased from Sigma-Aldrich (St. Louis, MO, USA). Cyanidin 3-O-glucoside was purchased from Sigma-Aldrich (Saint Quentin Fallavier, France). Carboxy-H_2_DCFDA was purchased from Invitrogen (Eugene, Oregon, USA). Lyophilized açaí fruit (*Euterpe oleracea* Mart.) was obtained from Liotécnica Alimentos LTDA (Embu, SP, Brazil).

### *Caenorhabditis elegans* Strains and Maintenance

The following strains were used in this study: Bristol N2 (wild-type; WT); LD1171, Is003 *(gcs-1::GFP)*; CL2166, dvls19[pAF15(*gst-4::GFP::NLS*)]; CF1553, muls84[pAD76(*sod-3::GFP*)]; VP198, kbIs5 [*gpdh-1p::GFP* + rol-6(su1006)]; TJ 356, zIs356[pGP30(*DAF-16::GFP*)+pRF4(*rol-6*)]; BA17, *fem-1(hc17)*; EU1, *skn-1(zu67) IV/nT1*; CF1038, *daf-16(mu86)*; AU3, *nsy-1(ag3)*; KU4, *sek-1(km4)*; VC8, *jnk-1(gk7)*; AM1, *osr-1(rm1)*; and MT2605, *unc-43(n498n1186)*. All *C. elegans* strains were maintained at 20°C on solid nematode growth medium (NGM) seeded with *E. coli* (OP50) as a food source according to Brenner [24]. The synchronization of worm cultures was achieved by hypochlorite treatment of gravid hermaphrodites.

### Human Umbilical Vein Endothelial Cells (HUVECs) Culture

HUVECs isolated previously by Boulanger *et al*. [25] were collected from the veins of umbilical cords obtained from Jeanne de Flandres Maternity (Lille University Hospital, France). Cells were cultured in M199 (2.5 µg/mL Fungizone, 100 U/mL penicillin, 100 µg/mL streptomycin, 2 mM L-glutamine and 150 mM Hepes) supplemented with 20% FBS. HUVECs were maintained in a humidified atmosphere containing 5% CO_2_ at 37°C.

### Açaí Aqueous Extract Preparation and Treatment

For all the experimental procedures, lyophilized açaí fruit was diluted with S basal solution and filter sterilized to obtain an aqueous extract (AAE). Control solution (S basal) or 100 mg/mL AAE was then mixed with an *E. coli* (OP50) pellet at OD 1 and seeded onto NGM plates. In experiments conducted with dead bacteria, NGM plates seeded with *E. coli* OP50, with or without 100 mg/mL AAE, were treated with 10 mM Kanamycin (KAN).

### Anthocyanin Quantification

Total anthocyanin was quantified by the pH differential method as described by Giusti *et al*. [26]. Diluted samples were added to 0.025 M chloride buffer (pH 1.0) and 4.0 M sodium acetate buffer (pH 4.5). After incubation in the dark for 30 min at room temperature, absorbances were determined simultaneously as absorption maxima for the visible light spectrum and at 700 nm (SP-220, Biospectro, PR, Brazil). The total anthocyanin content was expressed in mg of cyanidin-3-glucoside equivalent per 100 g of açaí powder. A molar absorptivity of 26,900 M^−1^ cm^−1^ and a molecular mass of 449.2 g/mol were used for cyanidin-3-glucoside.

The quantification of cyanidin 3-O-glucoside and cyanidin 3-O-rutinoside was performed in duplicate using a modified method from Gordon *et al*. [27]. Briefly, 0.25 g of sample was diluted in 3 mL of methanol/water/acetic acid (50∶49.5∶0.5, v/v/v). After shaking for 5 min, the sample was sonicated for 20 sec and centrifuged at 8,000 rpm for 10 min at 10°C. The supernatant was collected, and the pellet was re-extracted twice. The three supernatants were pooled, transferred into a 10 mL volumetric flask and diluted with methanol/water/acetic acid mix. After filtration through a 0.45 µm filter, each sample was analyzed by LC-MS/MS. Mass spectrometry analyses were carried out on a Thermo Scientific TSQ Quantum Discovery MAX triple-stage quadrupole mass spectrometer (Thermo Fisher Scientific, Courtaboeuf, France) with an electrospray ionization (ESI) probe coupled to an Accela HPLC system (Thermo Fisher Scientific, Courtaboeuf, France). The analytical separation was performed on a Symmetry Shield RP_18_ column, 150×2.1 mm, 3.5 µm (Waters, Guyancourt, France), at 20°C. The mobile phase was composed of 0.1% (v/v) formic acid in water (A) and 0.1% (v/v) formic acid in acetonitrile (B) and was eluted at a flow rate of 0.2 mL/min. The HPLC gradient started with 5% B for 20 min, reaching 30% B at 40 min and 95% B at 45 min. Mass spectra for the determination of anthocyanins were obtained by using positive ionization mode. Tandem MS analyses were performed in Selected Reaction Monitoring (SRM) mode. The specific transitions m/z 449.0 → m/z 287.1 and m/z 595.0 → m/z 287.1 were used for the detection and quantification of cyanidin 3-O-glucoside and cyanidin 3-O-rutinoside, respectively. As no stable isotopically labeled internal standard for anthocyanins is commercially available, the quantification was performed using the standard addition method and expressed as mg/100 g of dry matter.

### DPPH Radical Scavenging Assay

The DPPH Radical Scavenging Activity of AAE was determined as described by Brand-Willians *et al*. [28]. In short, 100 µL of açaí extract (1, 10 and 100 mg/mL) was added to 3.9 mL of 60 µM DPPH dissolved in 80% methanol. The mixture was homogenized and kept in the dark for 30 min at room temperature, after which the absorbance at 515 nm was determined (SP-220, Biospectro, PR, Brazil). A calibration curve was prepared using 2,5,7,8-tetramethylchroman-2-carboxylic acid (Trolox) in the concentration range 200–800 µM. The percentage of inhibition was determined according to the following equation: % Scavenging activity  =  (1 – Abs Sample 515/Abs Control 515) ×100. The DPPH radical scavenging assay was conducted twice.

### Body Length and Brood Size Assays

To measure body length, first-larval-stage animals (L1) were treated with control solution (S basal) or 100 mg/mL AAE until the third larval stage (L3) and then transferred to NGM plates with *E. coli* OP50 until the next day. Images were captured (Axio Imager Z2, Zeiss, NY, USA) of one-day-old animals, and body length was measured along the animal axis using NIH Image J software.

To determine total progeny production, ten fourth-larval-stage (L4) worms, previously treated with control solution (S Basal) or 100 mg/mL AAE since L1, were placed onto the *E. coli* OP50 lawn on individual NGM plates in the presence or absence of 100 mg/mL AAE. During the egg-laying period, nematodes were transferred onto new plates every 24 h for five days until the end of the reproductive period. The F1 progeny from each individual worm was counted after approximately two days. The total progeny numbers for each plate were calculated and divided by the number of animals [29]. Both experiments were conducted three times.

### Longevity

The longevity assay was performed with the adult sterile strain *fem-1(hc17)* as the wild-type strain to avoid progeny overgrowth in lifespan. Therefore, eggs were shifted to 25°C, the nonpermissive temperature for *fem-1(hc17)* fertility. Lifespan was scored every day after hermaphrodites completed the final larval molt, from the first day of adulthood (defined as t = 0) until death. We analyzed approximately 90 hermaphrodites treated with control solution (S basal) or 100 mg/mL AAE and divided into three NGM plates of 30 animals each. Animals were scored as dead if they displayed no spontaneous movement or failed to respond when prodded. Dead worms that displayed internally hatched progeny, an extruded gonad or desiccation caused by crawling off the agar were excluded from the data [21]. The longevity assay was conducted three times.

### Stress Resistance Assays

To evaluate oxidative stress resistance, N2 wild-type animals and *skn-1(zu67)*, *daf-16(mu86)*, *nsy-1(ag3)*, *sek-1(km4)*, *jnk-1(gk7)*, *osr-1(rm1)* and *unc-43(n498n1186)* mutants were treated with control solution (S basal) or 100 mg/mL AAE from L1 until L4 and then with 7.5 mM *tert*-butyl hydrogen peroxide (t*-*BOOH) in M9. To perform the oxidative stress resistance assay in dead bacteria, NGM plates seeded with *E. coli* OP50, with or without 100 mg/mL AAE, were treated with 10 mM KAN. Survival was measured at 3, 6, 9 and 12 h. We analyzed five wells, each with approximately ten worms, for each experimental group. Worms were prodded with a platinum wire and scored as dead if they displayed no pharyngeal pumping or movement [30].

Thermotolerance assays were performed with hermaphrodites on adult day 5, after the majority of egg-laying had ceased. Animals treated at 20°C with control solution (S basal) or 100 mg/mL AAE from L1 until adult day 5 were transferred onto 3-cm NGM agar plates supplemented as indicated above and then incubated at 35°C for 12 h. Survival was monitored at 6, 9 and 12 h and scored as the number of animals responsive to gentle touch as a fraction of the original number of animals on the plate. Animals that had died from desiccation on the sides of the plate were excluded [21]. To quantify the percent of motile worms under acute osmotic stress, animals were treated with control solution (S basal) or 100 mg/mL AAE for 68 h from L1 and then transferred to new plates containing 500 mM NaCl. The percentage of worms that moved outside a 7-mm circle was monitored at 15, 30 and 60 min. All experiments measuring oxidative stress resistance were conducted at least twice. The heat stress and motility under acute osmotic stress assays were each performed three times.

### Bacterial Growth Curve

*E. coli* OP50 growth was evaluated over 4 h in the presence of 100 mg/mL AAE. All OD readings at 600 nm were normalized to the OD of the control group at time zero. Bacterial growth was measured in three individual experiments.

### *In vivo* Measurement of ROS

ROS production was measured in *C. elegans* and HUVECs using the fluorescent probe 2′, 7′-dichlorofluorescein diacetate (H_2_DCFDA). ROS production in *C. elegans* was performed as described by Shi *et al*. [31]. Synchronized L1 animals were treated with control solution (S basal) or 100 mg/mL AAE for 48 h. L4 worms were incubated in 1 mL PBS containing 1 mM H_2_O_2_ for 2 h. Subsequently, the worms were washed twice and incubated in 0.5 mL PBS containing 50 µM H_2_DCFDA for 1 h. Thirty animals in experimental triplicates of each group were then transferred into the wells of a 96-well microtiter plate containing 200 µL PBS. The fluorescence quantification was carried out on a multilabel microplate Reader VICTOR *X*3 (Perkin Elmer, Massachusetts, USA) using excitation at 485 nm and emission at 535 nm.

ROS production in HUVECs was performed as described by Montiel-Dávalos *et al*. [32]. HUVECs were grown in 12-well plates to a density of 1×10^5^ cells per well. Confluent cells were then pretreated with or without 2.5 mg/mL AAE for 16 h, followed by incubation for 30 min with 10 µM H_2_DCFDA at 37°C in the dark. After this, the cells were washed with PBS and then treated with or without 0.25 mM H_2_O_2_ for 1 h. Cells were washed once and harvested in PBS. Fluorescence was detected with a flow cytometer using excitation at 488 nm and emission at 525 nm. In both *C. elegans* and HUVECs, the fluorescence of the control group was used to normalize the values from all other groups. The experiments were repeated at least four times.

### Quantification of Total Sulfhydryl (SH) Levels

To measure the levels of total SH groups, approximately 5,000 animals were treated with control solution (S basal) or 100 mg/mL AAE from L1 until L4 and then incubated with or without 5 mM t-BOOH for 1 h. The animals were then washed with M9 buffer and sonicated in 2-mL microcentrifuge tubes. The resulting homogenate was centrifuged, and the supernatant was collected, discarding cellular debris and intact worms. The total protein content was determined according to the method described by Lowry *et al*. [33] using bovine serum albumin (BSA) as a standard. The total and free serum sulfhydryl groups were estimated using Ellman's reagent according to Sedlak and Lindsay [34]. The protein-bound sulfhydryl groups were determined as the difference between the total and free sulfhydryl groups. The experiment was repeated twice.

### Reporter Gene Expression

Transgenic worms containing reporter genes were treated with control solution (S basal) or 100 mg/mL AAE for 48 h starting at L1, followed by the presence or absence of oxidative stress. The stress condition was 7.5 mM t-BOOH for 1 h for *gcs-1::GFP* and *gst-4::GFP* gene expression analysis and 10 mM t-BOOH for 1 h for *sod-3::GFP*. To analyze the subcellular localization of *DAF-16::GFP*, synchronized L1 transgenic worms were treated with control solution (S basal) or 100 mg/mL AAE for 48 h with or without subsequent 7.5 mM t-BOOH treatment for 1 h. Twenty worms from each group were mounted onto microscope slides coated with 1% agarose, anaesthetized with 0.5 mM sodium azide and capped with coverslips. Photographs were taken on a fluorescence microscope (Axio Imager Z2, Zeiss, NY, USA), and GFP fluorescence signals were measured using NIH Image J software. For *DAF-16::GFP*, expression patterns were classified as cytosolic, intermediate or nuclear. The experiment was conducted three times.

### Analysis of gene expression by qPCR

Synchronized L1 larvae were grown in plates containing *E. coli* OP50 bacteria resuspended in basal solution or AAE 100 mg/mL until L4 stage. Total RNA from worms was isolated using BRAZOL (LCG Biotecnologia, São Paulo, Brazil) according to manufacturer's instructions and cDNA was synthesized using High-Capacity cDNA Reverse Transcription Kits (Applied Biosystems). The qPCR was performed on a Applied Biosystems 7500 Real-Time PCR System (Applied Biosystems, Carlsbad, CA, USA) using a Power SYBR Green PCR master mix (Applied Biosystems). qPCR levels were normalized to the expression of *ama-1*, which encodes the large subunit of RNA polymerase II. The fold change was normalized to that observed in untreated *C. elegans* samples. Primers sequences for *ama-1*, *daf-16*, *gst-7*, *ctl-1*, *sod-3* and *osr-1* are listed in Table S1. The gene expression was analyzed in three individual experiments.

### Polyglutamine (PolyQ) Aggregation Quantification

Transgenic worms carrying the reporter gene *vha-6::Q44::YFP* were treated with control solution (S basal) or 100 mg/mL AAE since L1. Photographs of one-, four-, eight- and twelve-day-old animals were taken with a fluorescence microscope (Axio Imager Z2, Zeiss, NY, USA), and the numbers of aggregates were counted. The experiment was repeated three times.

### Proteasome Activity Quantification

*In vitro* 26S proteasome activity assays were performed as described by Kisselev and Goldberg [35]. Approximately 5,000 N2 wild-type animals were treated with control solution (S basal) or 100 mg/mL AAE for 48 h at L1. L4 worms were then harvested and sonicated. The lysates were centrifuged at 20,000×g for 20 min at 4°C. Protein extract was quantified using the QuantiPro BCA Assay Kit (Sigma Aldrich, St. Louis, MO, USA). For measuring the chymotrypsin-like activity of the proteasome, succinyl-Leu-Leu-Val-Tyr-4-methyl-coumaryl-7-amide (SLLVY-MCA) (Sigma-Aldrich, St. Louis, MO, USA) was used both in the presence or absence of 20 µM MG-132, a proteasome inhibitor. Enzyme kinetics were monitored in a temperature-controlled microplate reader VICTOR *X*3 (Perkin Elmer, Massachusetts, USA) every 15 min for 1 h at 37°C; the excitation and emission wavelengths were 380 and 460 nm, respectively. Proteasome activity was calculated as the difference between the total activity and the activity remaining in the presence of 20 µM MG-132. The proteasome activity quantification was conducted three times.

### Data Analysis

Statistical analysis was performed using GraphPad Prism version 5.00 for Windows (San Diego, CA). The results were plotted as the mean ± SEM (standard error of the mean) of at least two individual experiments. Data were subjected to the Kolmogorov-Smirnov test for normality. For data with a normal distribution, Student's *t* test was used to compare pairs of groups, whereas a one-way ANOVA followed by Tukey's post-test was used to compare three or more groups. Nonparametric data were analyzed using the Mann-Whitney test when comparing two groups and the Kruskal-Wallis test followed by Dunn's post-test for comparing three or more groups. All survival curves were analyzed by the Log-rank (Mantel-Cox) test. The statistical significance was determined as p<0.05.

## Results

### AAE phytochemical composition and antioxidant capacity *in vitro* and *in vivo*

We first characterized the composition of anthocyanins and the *in vitro* antioxidant capacity of our extract. AAE presents 31.0±2.4 mg/100 g of the total anthocyanins measured by the pH differential method. Cyanidin 3-O glucoside and cyanidin 3-O rutinoside are the two major anthocyanins in açaí pulp; AAE presents 8.8±0.9 mg/100 g of cyanidin 3-O glucoside and 8.7±0.9 mg/100 g of cyanidin 3-O rutinoside, as determined by LC-MS/MS analysis (Table 1). Açaí's polyphenolic compounds have already been associated with its *in vitro* antioxidant capacity [3], [36], [37]. We next determined the antioxidant capacity of 1, 10 and 100 mg/mL AAE by the DPPH radical scavenging activity method. We observed that AAE displays increasing *in vitro* antioxidant capacity in a dose-dependent manner (Table 2). At 100 mg/mL AAE, the highest concentration tested, the DPPH inhibition was 79.61%, which is equivalent to 800 µM Trolox, the reference standard, which shows 80.90% inhibition. Moreover, the percentage of DPPH inhibition for any concentration tested was not significantly altered when the extracts were mixed with *E. coli* OP50. Thus, we used AAE at 100 mg/mL with live *E. coli* OP50 for the subsequent experiments.

**Table 1:** Determination of anthocyanins present in açaí aqueous extract (AAE).

Compounds	Concentration (mg/100 g)
**Total monomeric anthocyanins** a	31.0±2.4
**Cyanidin 3-O glucoside** b	8.8±0.9
**Cyanidin 3-O rutinoside** b	8.7±0.6

The results are expressed as the mean ± SEM (standard error of the mean).

a Total monomeric anthocyanins measured by the pH differential method.

b Cyanidin 3-O glucoside and Cyanidin 3-O rutinoside determined by LC-MS/MS analysis.

**Table 2:** *In vitro* antioxidant activity of açaí aqueous extract (AAE) measured by DPPH assay.

	% inhibition (Mean ± SEM)			
AAE (mg/mL)	AAE	AAE + OP50	AAE + OP50 + KAN	AAE + KAN
1	5.07±0.75^a^	4.38±0.98^a^	−0.71±0.28^b^	0.10±0.16^b^
10	53. 30±5.61^a^	49.53±4.07^a^	18.55±2,18^b^	25.68±2.22^b^
100	79.61±3.33^a^	77.30±3.49^a^	73.46±4.54^a^	78.35±1.78^a^

DPPH, 2,2-diphenyl-1-picrylhydrazyl; Trolox, 6-hydroxy-2,5,7,8-tetramethylchroman-2-carboxylic acid; OP50, *E. coli* strain; KAN, Kanamycin.

Different subscript letters indicate significant differences by one-way ANOVA followed by Tukey's post-test.

### AAE does not alter *C. elegans* development and progeny

**Figure 1:**
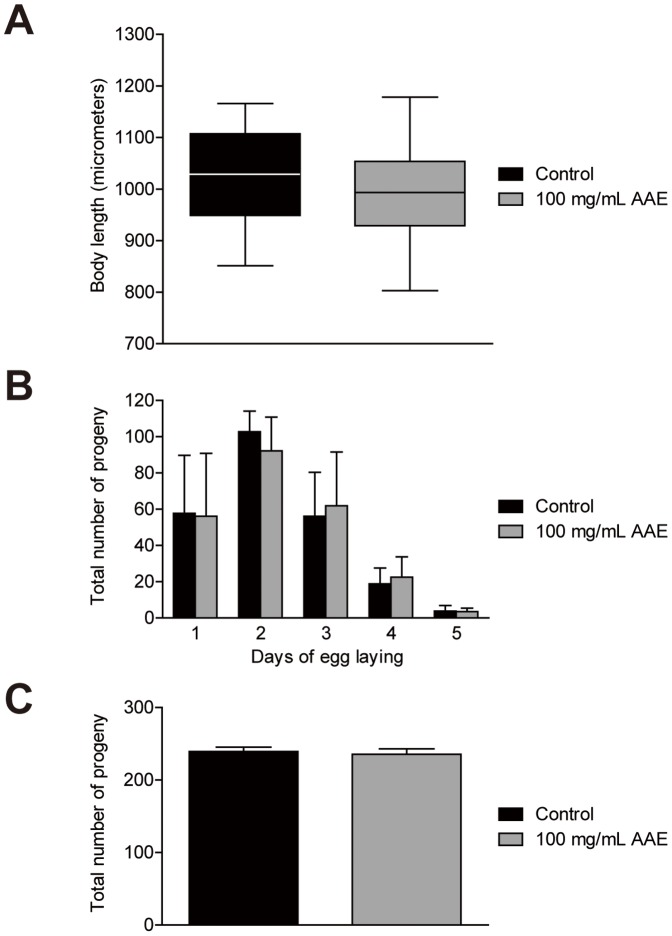
Effect of açaí aqueous extract (AAE) on wild-type *C. elegans* body length and progeny. **A**). L1 animals were treated with control solution (S basal) or 100 mg/mL AAE until L3 and then transferred onto NGM plates with OP50 until the next day. Images were captured of one-day-old animals, and body length was measured along the animal axis using NIH Image J software. There was no significant difference between groups, as determined by two-tailed Student's *t-*test. **B, C**) Progeny profiles were measured in animals treated with control solution (S basal) or 100 mg/mL AAE. Animals were transferred individually to NGM plates and moved daily until the end of the reproductive period. The results were plotted as the mean ± SEM for each day (B) and total final progeny (C). There was no significant difference between groups by a two-tailed Student's *t-*test.

As toxic compounds usually delay *C. elegans* development and progeny [38], we characterized the effect of AAE treatment on these two biological parameters. Body length was determined in one-day-old adults treated with or without 100 mg/mL AAE from L1 until L3 and then transferred to NGM plates with *E. coli* OP50. We did not observe a significant difference in body length between control (1020.0±11.6 µm) and AAE-treated animals (994.3±10.5 µm) (Figure 1A). To measure the total number of progeny, animals were treated with control solution (S basal) or 100 mg/mL AAE from L1 until the end of their reproductive period. There was no significant difference in the total number of progeny between control (239.4±6.1) and AAE-treated animals (235.6±7.5) (Figure 1B). In addition, there was no difference in the egg-laying profile between the two groups (Figure 1C). These results suggest that 100 mg/mL AAE is not toxic to *C. elegans*, as it does not interfere with development and progeny (Figure 1A, 1B and 1C).

### AAE increases resistance to oxidative and hyperosmotic stress conditions

**Figure 2:**
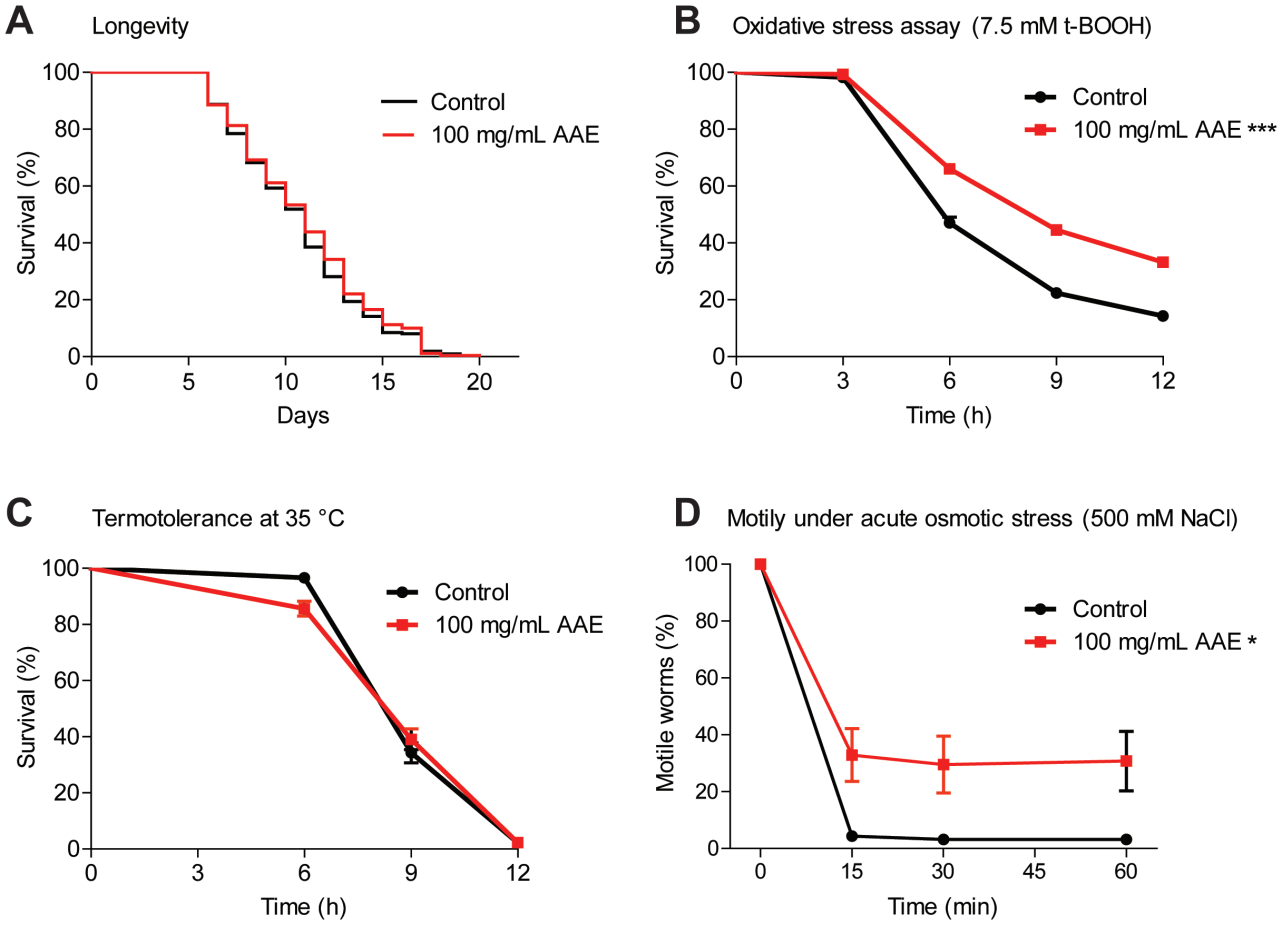
Effect of açaí aqueous extract (AAE) on *C. elegans* grown under normal and stress conditions. **A**) *fem-1(hc17)* mutants were treated at 25°C with control solution (S basal) or 100 mg/mL AAE beginning at L1. Surviving and dead animals were counted daily until all nematodes had died. Log-rank (Mantel-Cox) analysis showed no significant difference between the curves. **B**) Animals were treated with control solution (S basal) or 100 mg/mL AAE from L1 until L4 and then submitted to 7.5 mM t-BOOH in M9. The survival was measured at 6, 9 and 12 h. The survival curves show that AAE treatment increased *C. elegans* oxidative stress resistance. ***p<0.001 by the Log-rank (Mantel-Cox) test. **C**) Animals were treated with control solution (S basal) or 100 mg/mL AAE beginning at L1. After five days at 20°C, the animals were incubated at 35°C and survival was monitored at 6, 9 and 12 h. There was no significant difference between curves by the Log-rank (Mantel-Cox) test. **D**) Animals were treated with control solution (S basal) or 100 mg/mL AAE for 68 h beginning at L1 and then transferred to new plates containing 500 mM NaCl. The percentage of worms that moved outside a 7-mm circle was monitored at 15, 30 and 60 min. *p<0.05 by a two-tailed Student's *t-*test.

As AAE shows *in vitro* antioxidant activity and is not toxic to *C. elegans*, we next evaluated whether AAE treatment has a protective effect under normal and oxidative, heat and hyperosmotic stress conditions. To monitor longevity under normal conditions, *fem-1(hc17)* mutants grown at 25°C were treated with control solution (S basal) or 100 mg/mL AAE beginning at L1. AAE treatment had no effect on the *C. elegans* aging profile (p = 0.2997) (Figure 2A). Oxidative stress resistance assays were performed in wild-type animals treated with control solution (S basal) or 100 mg/mL AAE from L1 until L4 and then with 7.5 mM t-BOOH in M9. AAE showed increased oxidative stress resistance when compared to the control (p = 0.0002) (Figure 2B). The effect of AAE on heat tolerance was analyzed at 35°C in five-day-old wild-type animals with or without 100 mg/mL AAE treatment from L1. AAE did not protect *C. elegans* against heat stress when compared to the control group (p = 0.7388) (Figure 2C). Finally, we analyzed motility under acute osmotic stress at 500 mM NaCl. For the motility assay, L1 wild-type animals were treated with 100 mg/mL AAE for 68 h and then transferred to NGM plates with 500 mM NaCl. AAE treatment significantly increased motility under the osmotic stress condition (Figure 1D). After 1 h, 48.31% of the animals treated with 100 mg/mL AAE showed motility compared to 27.70% from the control group (p<0.05). We conclude that 100 mg/mL AAE protects against oxidative and hyperosmotic stress conditions but not under normal and heat stress conditions.

### The protective effect of AAE against oxidative stress is independent of its antimicrobial effect

**Figure 3:**
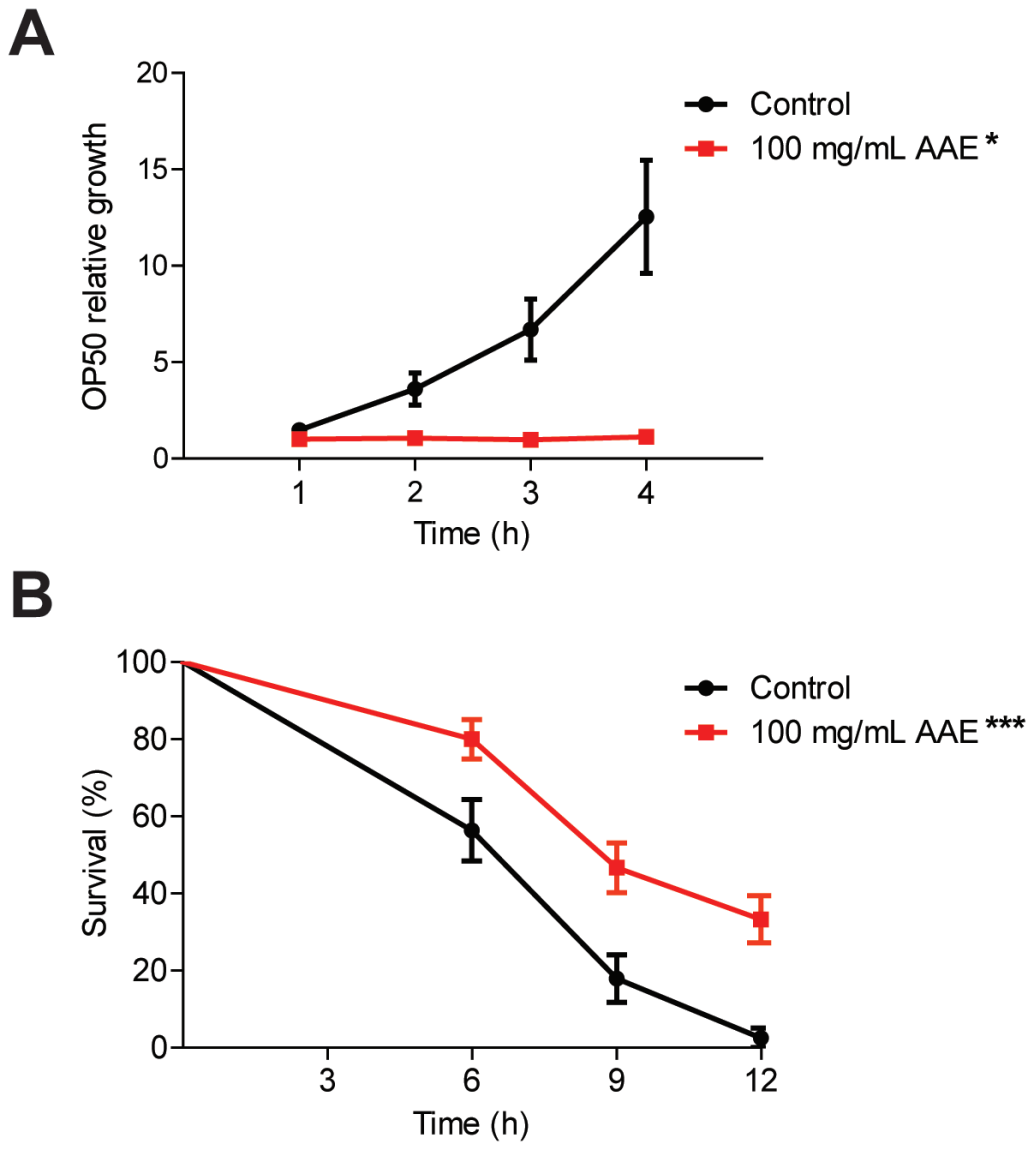
Effect of açaí aqueous extract (AAE) on the bacterial growth and oxidative stress resistance of wild-type *C. elegans* grown on dead bacteria. **A**) *E. coli* OP50 growth was evaluated over 4 h in the presence of 100 mg/mL AAE. The OD of the control group at time zero was used to normalize all other OD readings. * Treatment of 100 mg/mL AAE decreased bacteria growth at all times analyzed with p<0.05, determined by a two-tailed Student's *t-*test. **B**) Animals were treated with or without 100 mg/mL AAE, mixed with either *E. coli* OP50 or *E. coli* OP50 treated with 10 mM Kanamycin (KAN), from L1 until L4 and then submitted to 7.5 mM t-BOOH in M9. The survival was measured at 6, 9 and 12 h. The survival curves show that AAE treatment increased *C. elegans* oxidative stress resistance independent of its antibacterial effect. ***p<0.001 related to the respective control by the Log-rank (Mantel-Cox) test.

As bacteria play a role in *C. elegans* mortality and stress resistance [39], [40], we investigated whether the increased oxidative stress promoted by AAE could be a secondary response to a possible AAE antimicrobial property. First, we evaluated *E. coli* OP50 growth, measuring the OD over 4 h in the presence or absence of 100 mg/mL AAE. AAE treatment decreased bacteria growth at all times analyzed (Figure 3A). Because we observed that 100 mg/mL AAE exerted an antimicrobial effect, we repeated the oxidative stress resistance assay in animals treated with AAE on dead bacteria. We observed that wild-type animals treated with 100 mg/mL AAE presented increased survival on 7.5 mM t-BOOH compared to controls (p<0.0001) (Figure 3B). These results show that AAE treatment does not increase *C. elegans* oxidative stress resistance through an antimicrobial property.

### AAE improves redox status in *C. elegans* and HUVECs under oxidative stress conditions

**Figure 4:**
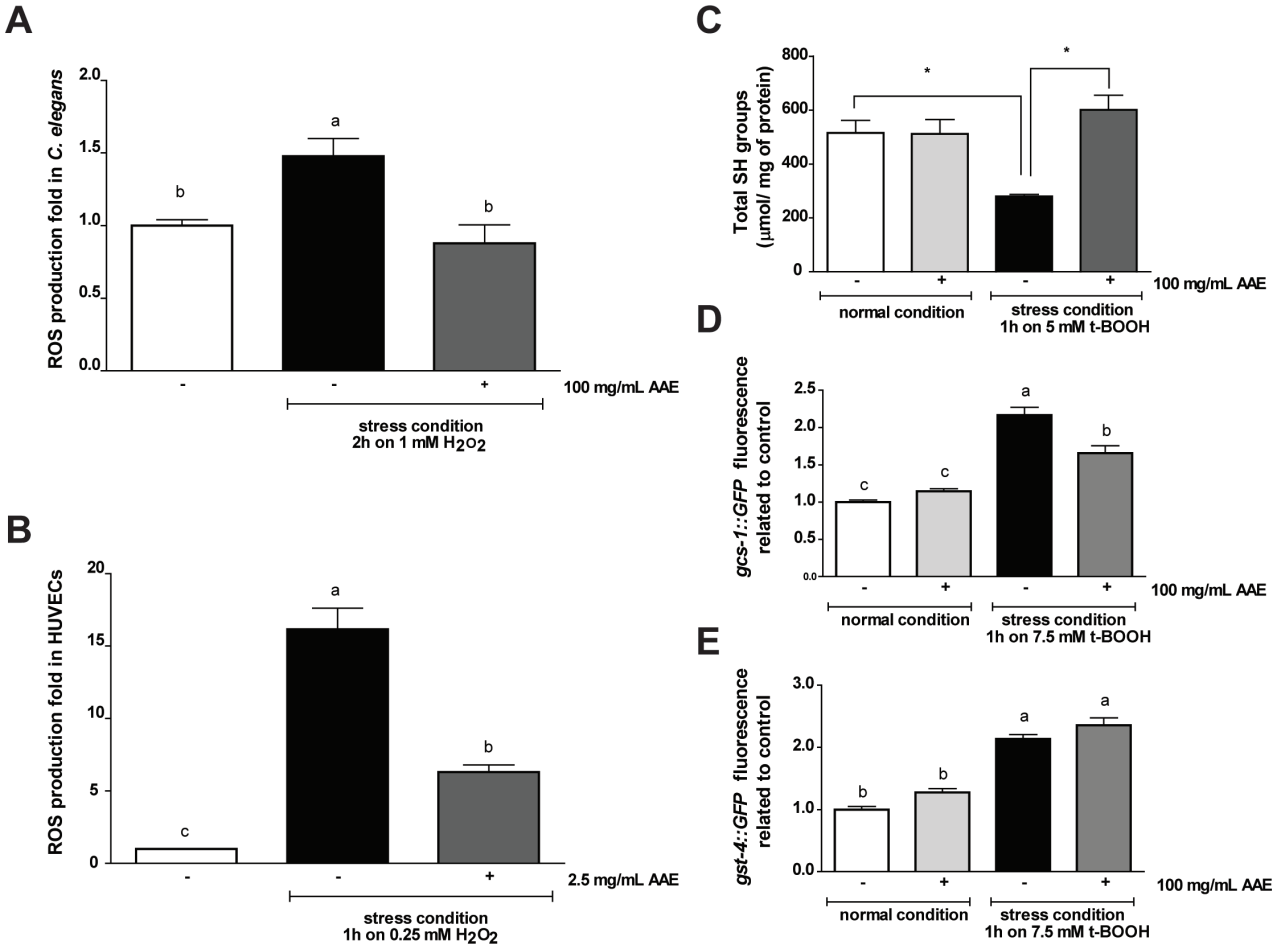
Effect of açaí aqueous extract (AAE) on redox status in wild-type *C. elegans* and HUVECs. **A**) *C. elegans* was treated with control solution (S basal) or 100 mg/mL AAE for 48 h and then submitted to the presence or absence of 1 mM H_2_O_2_ for 2 h. The results are expressed as H_2_DCFDA fluorescence relative to the untreated control. **B**) HUVECs were treated with or without 2.5 mg/mL AAE for 16 h and then incubated in 0.25 mM H_2_O_2_ for 1 h. The fluorescence was measured by flow cytometry. The results are expressed as H_2_DCFDA fluorescence relative to the untreated control. Different letters indicate significant differences by one-way ANOVA followed by Tukey's post-test. **C**) To measure the levels of total SH groups, animals were treated with control solution (S basal) or 100 mg/mL AAE from L1 until L4 and then incubated with or without 5 mM t-BOOH for 1 h. *p values were determined by a two-tailed Student's *t-*test, and groups were significantly different when p<0.05 in *C. elegans*. Transgenic worms containing reporter genes were treated with control solution (S basal) or 100 mg/mL AAE for 48 h beginning at L1 and then with or without the oxidative stress condition. After a 1-h hour recovery period, photographs were taken on a fluorescence microscope. For (**D**) *gcs-1::GFP* and (**E**) *gst-4::GFP* animals, GFP fluorescence signals were measured using NIH Image J software. Different letters correspond to significant differences by the Kruskal-Wallis test followed by Dunn's post-test.

Potential antioxidants can act directly to neutralize reactive oxygen species (ROS) and thus prevent the cellular response to increased oxidative stress to combat and remove ROS. To evaluate the capacity of AAE to neutralize ROS *in vivo*, we assessed ROS production in two experimental models, *C. elegans* and HUVECs, using the H_2_DCFDA fluorescent probe. We did not detect an effect of AAE treatment on basal ROS levels in either *C. elegans* or HUVECs (data not shown). We observed a significant increase in fluorescence intensity in worms (Figure 4A) and cells (Figure 4B) treated with their respective stress conditions. However, AAE treatment significantly reduced intracellular ROS accumulation in *C. elegans* under stress conditions (p<0.01) (Figure 4A). In HUVECs, the stress condition increased ROS production by 16-fold relative to the control, and AAE diminished this increase by up to 6-fold (p<0.001) (Figure 4B).

As AAE exhibited an *in vivo* antioxidant capacity, we next evaluated whether AAE would improve redox status in wild-type *C. elegans*. We assessed the levels of total sulfhydryl (SH) groups in animals treated with control solution (S basal) or 100 mg/mL AAE from L1 until L4. The treatment of *C. elegans* with 100 mg/mL of AAE for 48 h did not increase the levels of total protein sulfhydryl groups (515.97±32.73 *vs*. 512.2±52.75 µmol/mg of protein). The stress condition significantly decreased the levels of SH groups (515.97±32.73 *vs*. 279.73±5.51 µmol/mg of protein) (p<0.05). However, when the worms were first exposed to AAE and then submitted to the stress condition with t-BOOH, this pretreatment was able to prevent the reduction of protein sulfhydryl group levels induced by t-BOOH (601.18±38.25 *vs*. 279.73±5.51 µmol/mg of protein) (p<0.05) (Figure 4C).

To correlate the *in vivo* antioxidant effect of AAE treatment with a molecular response in *C. elegans*, we analyzed the gene expression of γ-glutamyl cysteine synthetase-1 (*gcs-1*) and glutathione-s-transferase-4 (*gst-4*). GCS-1 is the limiting enzyme of glutathione (GSH) synthesis and is expressed at high levels in the intestine in response to oxidative stress but at low levels under normal conditions in an SKN-1-dependent manner [41]. *gst-4::GFP* is expressed primarily in the muscles and the hypodermis under normal conditions, and is increased in response to a variety of oxidative stress treatments [42], [43]. Transgenic worms containing reporter genes were treated with control solution (S basal) or 100 mg/mL AAE for 48 h beginning at L1 and either in the presence or absence of oxidative stress. The fluorescence signals of *gcs-1::GFP* and *gst-4::GFP* animals treated with 100 mg/mL of AAE for 48 h were not significantly increased compared to the control group (Figure 4D–E). Exposure to 7.5 mM t-BOOH for 1 h increased *gcs-1::GFP* and *gst-4::GFP* expression, and AAE significantly prevented the upregulation of *gcs-1::GFP* (Figure 4D) but not *gst-4::GFP* (Figure 4E). Taken together, these results suggest that AAE functions as a direct antioxidant through the removal of ROS, preventing the decrease in SH groups mediated by the stress condition.

### Increased oxidative stress resistance induced by AAE treatment depends on DAF-16 and OSR-1/UNC-43/SEK-1

Certain flavonoids protect against oxidative stress either directly, by radical scavenging, or indirectly, by inducting antioxidant enzymes and thus increasing the stress resistance of the organism. It has been shown in *C. elegans* that phytochemicals and plant extracts activate several stress response pathways [21], [23], [44]. To verify whether, in addition to its direct role, AAE protection under oxidative stress could also require these pathways, we performed the oxidative stress resistance assay in wild-type and mutant animals treated with 100 mg/mL AAE or control solution (S basal).

**Figure 5:**
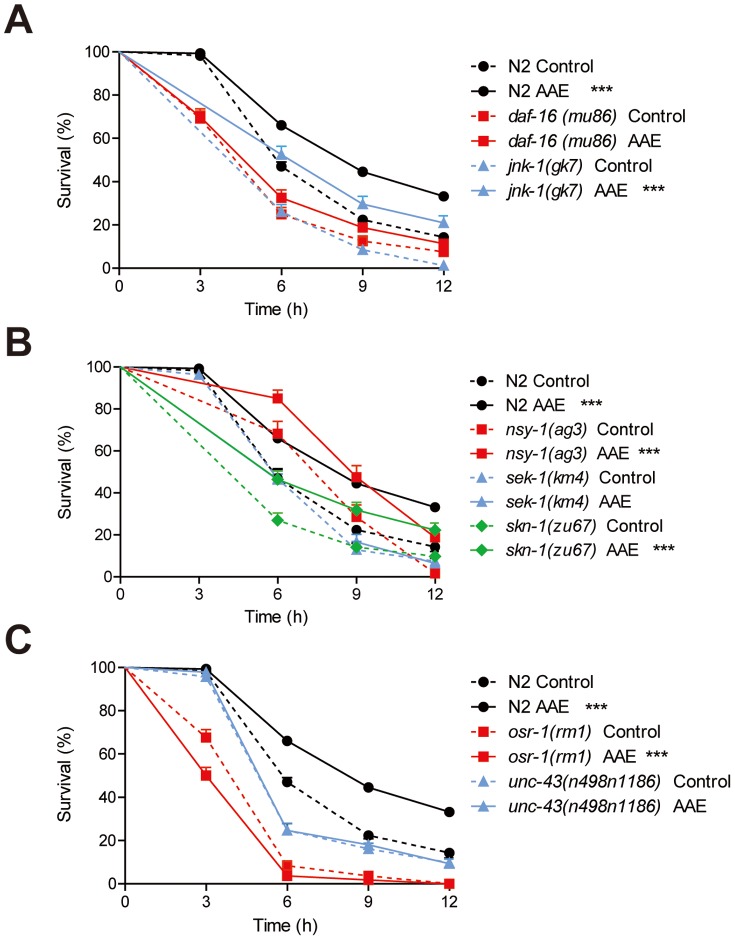
Contribution of genetic background to oxidative stress resistance induced by açaí aqueous extract (AAE) treatment. Animals were treated with 100/mL AAE or control solution (S basal) from L1 until L4 and then submitted to 7.5 mM t-BOOH in M9. Survival was measured at 3, 6, 9 and 12 h. **A**) Survival curves for transcription factor *daf-16 (mu86)* and JNK MAPK pathway *jnk-(gk7)* mutants. **B**) Survival curves for *skn-1(zu67)* and p38 MAPK pathway, *nsy-1(ag3)*, *sek-1(km4)* mutants. **C**) Survival curves for osmotic stress resistance pathway, *unc-43(n498n1186)* and *osr-1(rm1)* mutants. *p<0.05 and ***p<0.001 by the Log-rank (Mantel-Cox) test (see Table 3 for more details).

DAF-16/FOXO, the downstream target of insulin-like signaling in *C. elegans*, is a transcription factor required for both lifespan regulation and stress resistance [45], [46]. The nuclear translocation of DAF-16 is positively regulated by c-Jun N-terminal kinase (JNK-1) in parallel with insulin-like signaling [46]. We therefore investigated whether oxidative stress resistance induced by AAE treatment depends on JNK-1/DAF-16 signaling. AAE treatment did not increase *daf-16(mu86)* mean survival (p = 0.1782), whereas it increased the mean survival of *jnk-1(gk7)* (p<0.0001) (Figure 5A; Table 3). These results suggest that AAE may increase oxidative stress resistance via DAF-16 independently of JNK-1.

**Table 3:** Effect of açaí aqueous extract (AAE) on the oxidative stress resistance of wild-type and mutant *C. elegans*.

	Median survival (h)			n^a^	
	Control	Treated	P *vs*. Control (log-rank)^b^	Control	Treated
**N2**	7.4	8.0	<0.0001	665 (12)	669 (12)
***osr-1(rm-1)***	5.4	4.7	0.0009	167 (3)	166 (3)
***unc-43(n498n1186)***	6.6	6.7	0.7320	186 (3)	183 (3)
***daf-16(mu86)***	5.7	6.0	0.1782	185 (3)	160 (3)
***jnk-1(gk7)***	7.0	7.5	<0.0001	165 (3)	162 (3)
***nsy-1(ag3)***	8.9	9.5	0.0005	63 (2)	80 (2)
***sek-1(km4)***	7.3	7.6	0.7483	109 (2)	108 (2)
***skn-1(zu67)***	6.7	7.3	0.0002	163 (3)	157 (3)

a Total number of hermaphrodites analyzed. The number in parentheses indicates the number of independent trials.

b Comparisons were performed using the Log-rank (Mantel-Cox) test.

SKN-1/Nrf is a transcription factor that promotes the expression of antioxidant or detoxification enzymes, thus increasing stress resistance and longevity in *C. elegans* [19], [30]. Under oxidative stress, SKN-1 functions depend on p38 MAPK signaling through the NSY-1/SEK-1/PMK-1 pathway [47]. We measured the survival of *nsy-1(ag3)*, *sek-1(km4)* and *skn-1(zu67)* mutants treated with AAE. AAE treatment prolonged the mean survival of *nsy-1(ag3)* (p = 0.0005) and *skn-1(zu6)* (p = 0.0002) but not *sek-1(km4)* (p = 0.7483) (Figure 5B; Table 3). These results suggest that the AAE antioxidant effect does not depend on SKN-1, and may act via the p38 pathway, but only through SEK-1.

In *C. elegans*, survival under hyperosmotic stress requires the OSR-1/UNC-43/SEK-1 stress response pathway [48]. We also tested whether the protective effect of AAE treatment under oxidative stress conditions depends on OSR-1 and UNC-43. AAE treatment did not extend the mean survival of *unc-43(n498n1186)* (p = 0.7320) (Figure 5C, Table 3). In *osr-1(rm1)* mutants, AAE treatment reduced the mean survival under oxidative stress (p = 0.0009) (Figure 5C, Table 3). These findings suggest that AAE modulates oxidative stress resistance through OSR-1 and two of its downstream effectors: UNC-43 and SEK-1.

### AAE treatment induces *ctl-1* and *gst-7* expression in a DAF-16-dependent manner

**Figure 6:**
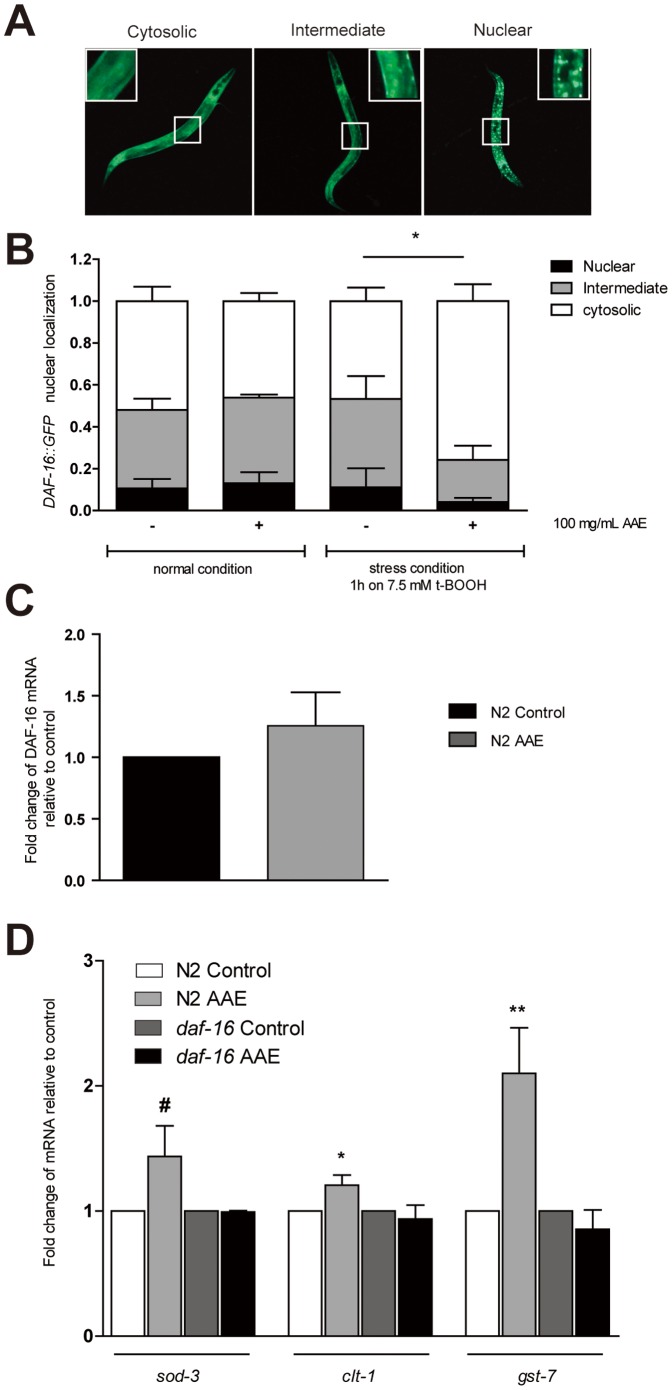
Effect of açaí aqueous extract (AAE) on *DAF-16::GFP* nuclear localization and the expression of its stress-inducible targets. Transgenic worms containing reporter genes were treated with control solution (S basal) or 100 mg/mL AAE for 48 h from L1 and then with or without the oxidative stress condition. **A, B**) *DAF-16::GFP*, worms were classified as cytosolic, intermediate or nuclear according to their subcellular distribution of DAF-16. **C**) mRNA level of *daf-16* in wild-type animals treated or not with 100 mg/mL AAE for 48 h. **D**) mRNA levels of DAF-16 stress-inducible genes *sod-*3, *ctl-*1 and *gst-7* in wild-type and *daf-16* animals treated or not with 100 mg/mL AAE. #p = 0.07, *p<0.05, **p<0.01 by a two-tailed Student's *t-*test.

As AAE treatment increased oxidative stress resistance in *C. elegans* through DAF-16, we investigated whether AAE treatment activates DAF-16 nuclear translocation under normal and stress conditions. The results showed that under normal growth conditions, AAE treatment did not induce DAF-16::GFP nuclear translocation (Figure 6A–B). Moreover, the AAE treatment did not alter *daf-16* mRNA levels in wild-type animals (Figure 6C). The stress condition used in this study (7.5 mM t-BOOH for 1 h) also did not increase the fraction of animals showing DAF-16::GFP nuclear localization (Figure 6B). However, under stress conditions, AAE treatment significantly reduced DAF-16::GFP nuclear localization (Figure 6B). One possible explanation for this finding is that AAE modifies DAF-16 to increase its activity but not its concentration. To further test this hypothesis, we measured the expression of the DAF-16 stress target genes after AAE treatment in wild-type animals and *daf-16* mutants (Figure 6D). The results showed that the transcripts levels of *ctl-1* and *gst-7* were upregulated in wild-type animals treated with AAE in a manner that was dependent upon *daf-16* (Figure 6D). However, AAE treatment did not induce neither *sod-3* mRNA (Figure 6D) nor *sod-3::GFP* expression (Figure S1). Taken together, these results suggest that AAE treatment increases oxidative stress resistance by activating antioxidant genes via DAF-16.

### AAE increases polyglutamine protein aggregation and decreases proteasome activity

OSR-1 is a secreted protein that couples with SEK-1/MAPKK through UNC-43/CaMKII to promote resistance to osmotic stress [48]. A loss of OSR-1 function constitutively activates *gpdh-1* expression and glycerol accumulation [49]. Glycerol replaces inorganic ions in the cytoplasm and functions as a chemical chaperone that aids in the refolding of misfolded proteins [50].

**Figure 7:**
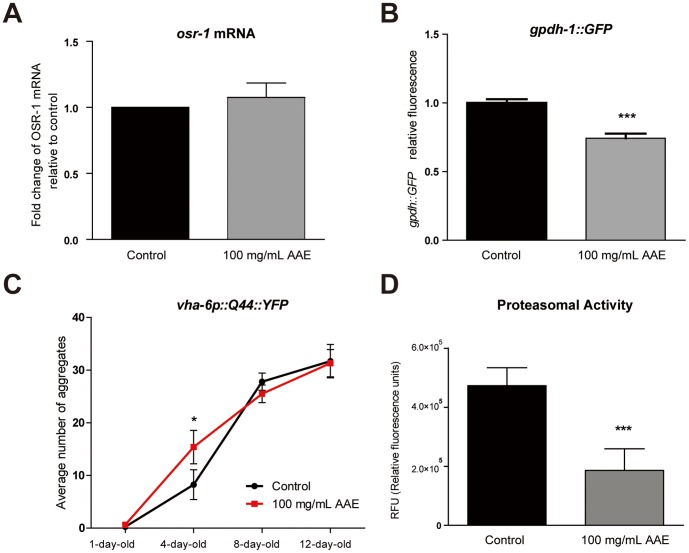
Effect of açaí aqueous extract (AAE) on protein homeostasis. **A**) mRNA level of *osr-1* in wild-type animals treated or not with 100 mg/mL AAE for 48 h. **B**) Transgenic worms carrying the reporter gene *gpdh-1::GFP* were treated or not with 100 mg/mL AAE for 48 h and then photographs were taken on a fluorescent microscope. GFP fluorescence signals were measured using NIH Image J software. ***p<0.001 by a two-tailed Student's *t-*test. **C**) Transgenic worms carrying the reporter gene *vha-6::Q44::YFP* were treated with control solution (S basal) or 100 mg/mL AAE starting at L1. Photographs of one-, four-, eight- and twelve-day-old animals were taken on a fluorescence microscope, and the numbers of aggregates were counted. *p<0.05 by a one-tailed Student's *t-*test. **D**) Animals were treated with 100 mg/mL AAE or control solution (S basal) from L1 until L4. Proteasome chymotrypsin-like activity was monitored by SLLVY-MCA digestion in L4 worm extracts containing equal amounts of total protein. ***p<0.001 by a two-tailed Student's *t-*test.

It is possible that AAE increases both oxidative and osmotic stress resistance by directly blocking OSR-1 activity and inducing *gpdh-1* expression. To test this hypothesis, we measured *osr-1* mRNA levels and *gpdh-1::GFP* expression in animals treated or not with 100 mg/mL AAE for 48 h. The AAE treatment did not modify *osr-1* gene expression under normal conditions (Figure 7A) and reduced *gpdh-1::GFP* expression (Figure 7B). Nevertheless, AAE treatment could interfere in OSR-1 activity which is coupled with the pathway UNC-43/SEK-1 in order to promote oxidative stress resistance.

Alternatively, AAE may function as a mild stressor, increasing hypertonic stress and/or protein homeostasis. High ionic strength is a well-known disruptor of protein secondary structure that can increase protein aggregation [51]. To test the hypothesis that AAE could increase hypertonic strength, we quantified aging-induced polyglutamine protein aggregation in *vha-6::Q44::YFP* transgenic worms. Q44 animals exhibit a diffuse fluorescence throughout the intestine until they reach adulthood, when focal fluorescent aggregates gradually increase. At one-day-old, Q44::YFP aggregation was not different between the control and 100 mg/mL AAE-treated groups (Figure 7C), whereas four-day-old Q44::YPF adult animals treated with 100 mg/mL AAE had approximately twice as many aggregates as control animals (p<0.05) (Figure 7C). Although the average number of aggregates increased with age, the AAE treatment had no effect on Q44::YFP aggregation in adult worms at eight- and twelve-day-old (Figure 7C). This result suggests that AAE treatment accelerates the aggregation formation at the young adult stage. To evaluate whether AAE treatment impairs protein homeostasis, we measured proteasome activity in animals treated with control solution (S basal) from L1 until L4. Proteasome chymotrypsin-like activity was monitored by SLLVY-MCA digestion in L4 worm extracts containing equal amounts of total protein. AAE decreased proteasome degradation activity by 2.5-fold relative to the controls (p<0.05) (Figure 7D). These results suggest that AAE may increase oxidative and osmotic stress resistance via increased ionic strength and/or impairment of protein homeostasis.

## Discussion

Açaí (*Euterpe oleracea* Mart.) has been highly praised in recent few years for having a wide range of health-promoting and therapeutic benefits due to its nutritional value and high levels of polyphenolic compounds, especially anthocyanin and proanthocyanidin. Although a great number of studies have reported that açaí treatment *in vitro* provides antiproliferative, anti-inflammatory, antioxidant and cardioprotective effects [4], *in vivo* studies are still lacking. Here, we employed an *in vivo* approach to investigate the antioxidant properties of açaí and its underlying mode of action by using the model organism *C. elegans*. Our results reveal that açaí protects against oxidative stress through both direct and indirect mechanisms. AAE increases resistance to oxidative and osmotic stress independently of any effect on reproduction, development and bacterial growth. AAE treatment directly reduces ROS production and prevents SH level reduction and *gcs-1* activation under oxidative stress conditions. Oxidative stress resistance is also indirectly mediated by AAE through DAF-16 and the OSR-1/UNC-43/SEK-1 osmotic stress pathway.

Many dietary polyphenols have antioxidant activity, and this activity is generally attributed to their ability to directly neutralize reactive oxygen and nitrogen species. The findings presented here support a direct mechanism of action for AAE through the reduction of ROS production induced by hydrogen peroxide (H_2_O_2_) in *C. elegans* and HUVECs. Many authors have already demonstrated that dietary supplementation with polyphenols and phytochemicals significantly decreases ROS production under stress conditions in *C. elegans*. For example, the flavonoids epicatechin, quercetin, rutin and G*inkgo biloba* extract EGb761 reduce the ROS accumulation induced by thermal stress [52]–[54]. Similarly, treatment with myricetin, a flavonoid commonly used as a natural chemopreventive, also reduces ROS accumulation in wild-type animals exposed to H_2_O_2_ [55]. Conversely, Guha *et al*. [23] demonstrated that cranberry extract (CBE) supplementation was not effective in reducing ROS levels in worms exposed to paraquat or in protecting worms exposed to oxidative stress.

**Figure 8:**
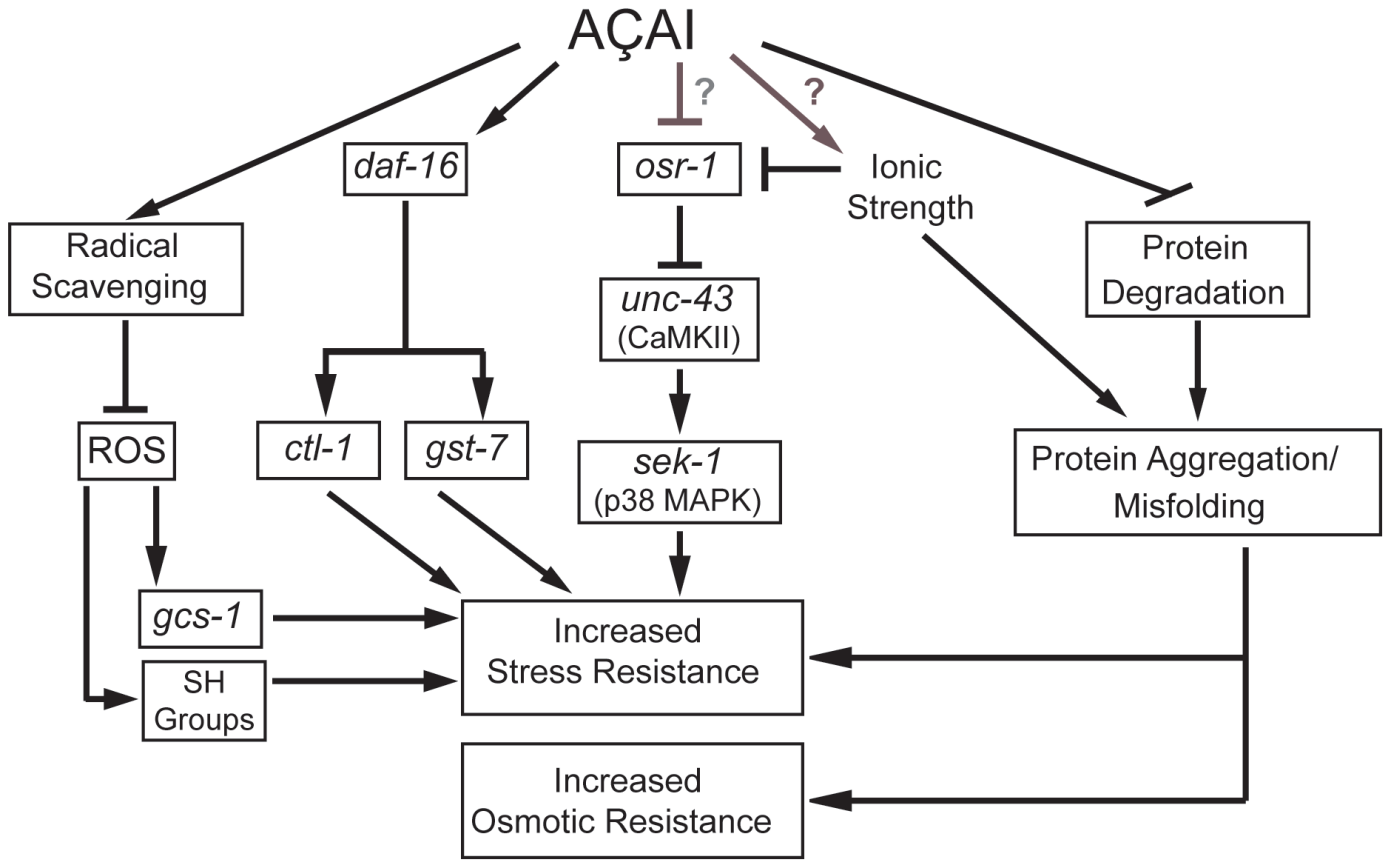
Hypothetical model of the mode of action of açaí aqueous extract (AAE) on *C. elegans*. Text marked within a rectangle represents modulators or data observed experimentally in this manuscript. AAE modulates oxidative stress resistance by direct and indirect mechanisms. AAE removes ROS directly and prevents *gcs-1* activation and SH level reduction which in turn increases oxidative stress resistance. AAE also promotes oxidative stress resistance indirectly through DAF-16/OSR-1/UNC-43/SEK-1. In addition, AAE increases osmotic stress resistance, possibly as a result of impaired protein homeostasis and/or increased ionic strength.

Many antioxidants can also increase oxidative stress resistance by inducing the transcription of cytoprotective proteins. Flavonoids and phytochemicals have been shown to increase the expression of protective genes *in C. elegans* such as *gst-4* [42], *gcs-1* [56] and *sod-3* [31]. In our work, AAE treatment did not alter the expression of *gst-4*, *gcs-1* or *sod-3* under normal conditions, but it prevented *gcs-1* activation under oxidative stress conditions. Moreover, AAE treatment prevented SH level reduction under oxidative stress conditions. Similarly, GSH levels in epicatechin-treated worms under thermal stress were restored to normal levels [52]. These findings support a direct mode of action for AAE. These results can be partially explained by the high levels of polyphenols present in açaí. In this scenario, the radical-scavenging properties of AAE's polyphenols, demonstrated both *in vitro* and *in vivo*, directly neutralize ROS produced under stress condition. The depletion of sulfhydryl groups, the most abundant and important nonenzymatic defense molecules, especially GSH, reflects intracellular oxidation. Thus, a reduction of the cellular stress environment promoted by AAE is able to avert the oxidation of sulfhydryl groups and the activation of *gcs-1*, the limiting enzyme of glutathione (GSH) synthesis (Figure 8).

We have demonstrated that AAE treatment also increases acute osmotic stress resistance in *C. elegans*. This activity can be explained not only by the radical-scavenging properties of AAE but also by an indirect activity through the activation of protective signaling pathways. Our genetic analysis indicates that stress resistance mediated by AAE is dependent on the DAF-16 and OSR-1/UNC-43/SEK-1 osmotic stress pathways.

The DAF-16/FOXO transcription factor is regulated by the insulin signaling pathway and is considered a key regulator of many important biological processes, including lifespan, metabolism and stress responses [57]. The nuclear translocation of DAF-16 and *sod-3* expression were reported for different extracts and polyphenols such as cranberry extract [23] and *Monascus*-fermented dioscorea [31], quercetin [58], myricetin and kaempferol [59]. Our results showed that AAE treatment does not increase oxidative stress resistance in *daf-16(mu86)* mutant animals, suggesting that AAE treatment protects against oxidative stress in a DAF-16-dependent manner. Nevertheless, AAE treatment does not increase DAF-16 nuclear localization or *sod-3* expression under normal conditions. One possible explanation for the requirement of DAF-16 on AAE-induced oxidative stress resistance without nuclear localization is that AAE might modify DAF-16 to increase its activity but not its concentration. Furthermore, DAF-16 activation by AAE in the nucleus might upregulate specific subsets of genes other than *sod-3*. In fact, AAE treatment increased the gene expression of *ctl-1* and *gst-7* in a DAF-16-dependent manner.

OSR-1 is a master regulator of *C. elegans* survival in hyperosmotic environments [48]; OSR-1 couples with SEK-1/MAPKK through UNC-43/CaMKII to promote resistance to chronic osmotic stress. The requirement of UNC-43 has been reported for the extended lifespan induced by blueberry [21], cranberry [23] and quercetin [44]. However, the prolongevity effect induced by these phytochemicals depends differently on OSR-1 and SEK-1. For example, SEK-1, but not OSR-1, is required for quercetin-induced longevity [44], whereas OSR-1, but not SEK-1, is required for cranberry-induced longevity [23]. Our genetic study indicates that OSR-1 is required for AAE-increased oxidative stress resistance as well as UNC-43 and SEK-1. Wilson *et al*. [21] found that blueberry polyphenol-induced longevity is dependent on the OSR-1/UNC-43/SEK-1 pathway. These results suggest that the OSR-1/UNC-43/SEK-1 pathway is a key target for anthocyanins, the predominant polyphenols present in açaí and blueberry.

Notably, prolongevity and thermotolerance are common features observed in *C. elegans* exposed to phytochemical interventions [21], [23], [44], [54]. The ability of these phytochemicals to increase resistance to heat stress may be due to their similar polyphenol content. Despite this similarity, neither of these effects were observed in the animals treated with AAE. Although blueberry and açaí extracts have similar polyphenol content, other bioactive metabolites are found in açaí extract. These compounds include peonidin 3-rutinoside and peonidin 3-glucoside, cyanidin 3-arabinosylarabinoside, cyanidin 3-arabinoside and cyanidin 3-acetyl hexose [2], [60]. As a number of experiments have shown that different phytochemicals may interact and synergize to exert their biological functions, it is not unexpected that AAE treatment does not increase thermotolerance in *C. elegans*.

Guha *et al*. [23] observed that CBE treatment decreases osmotic stress resistance in *C. elegans*. The authors explained this susceptibility through a depression of CaMKII/p38 MAPK signaling by CBE, based on the observation that *osr-1* mRNA is upregulated by CBE treatment. *osr-1* upregulation by flavonoids was also demonstrated by Xue *et al*. [61]. In our study, we demonstrated that AAE treatment increases acute osmotic stress resistance in *C. elegans*. However, the AAE treatment did not modify *osr-1* gene expression under normal conditions and reduced *gpdh-1::GFP* expression. These results did not support our hypothesis that the mechanism of AAE-increased osmotic stress resistance requires neither *osr-1* downregulation nor glycerol synthesis.

As stress resistance mediated by AAE treatment was dependent on OSR-1, we hypothesize that açaí might act directly by blocking OSR-1 protein activity or may function as a mild stressor (Figure 8). OSR-1 inhibition by AAE in turn activates UNC-43 and SEK-1 and consequently promotes oxidative stress resistance. If AAE treatment generates a hypertonic environment, we could expect increased protein aggregation and proteasomal activity. In agreement with our hypothesis, we observed that AAE treatment increased the number of Q44::YFP protein aggregates. However, proteasomal activity was significantly reduced, which might also contribute to the increased polyglutamine aggregation.

Our study highlights the *in vivo* antioxidant and stress resistance properties of AAE, supporting previous *in vitro* experiments on cultured cells and short-term rodent studies. This work also helps to reveal the underlying molecular mechanisms by which AAE modulates stress responses. We propose that AAE modulates oxidative stress resistance by direct and indirect mechanisms. AAE removes ROS directly and prevents *gcs-1* activation and SH level reduction, which in turn protects against oxidative stress. AAE also promotes oxidative stress resistance indirectly through DAF-16 and OSR-1/UNC-43/SEK-1. In addition, AAE increases osmotic stress resistance, possibly as a result of increased ionic strength and/or protein homeostasis impairment (Figure 8). Regardless of the specific mechanism involved, our findings indicate that the natural compounds available in AAE can improve the antioxidant status of a whole organism under certain conditions.

## Supporting Information

Figure S1 **Effect of açaí aqueous extract (AAE) on** ***sod-3::GFP*** **expression.** Transgenic worms carrying the reporter gene *sod-3::GFP* were treated with control solution (S basal) or 100 mg/mL AAE for 48 h from L1 and then with or without the oxidative stress condition. After a 1-h recovery period, photographs were taken on a fluorescence microscope. GFP fluorescence signals were measured using NIH Image J software. Different letters correspond to significant differences by the Kruskal-Wallis test followed by Dunn's post-test. AAE treatment alone nor the exposure to 10 mM t-BOOH for 1 h significantly upregulated *sod-3::GFP* expression.
(TIF)

Table S1 **List of primers for qPCR.**
(DOCX)
