# Supplementary figures and images for: Açaí (Euterpe oleracea Mart.) Modulates Oxidative Stress Resistance in Caenorhabditis elegans by Direct and Indirect Mechanisms
Source: PLoS One. 2014 Mar 3;9(3):e89933. doi: 10.1371/journal.pone.0089933 (PMC3940722; doi:10.1371/journal.pone.0089933)

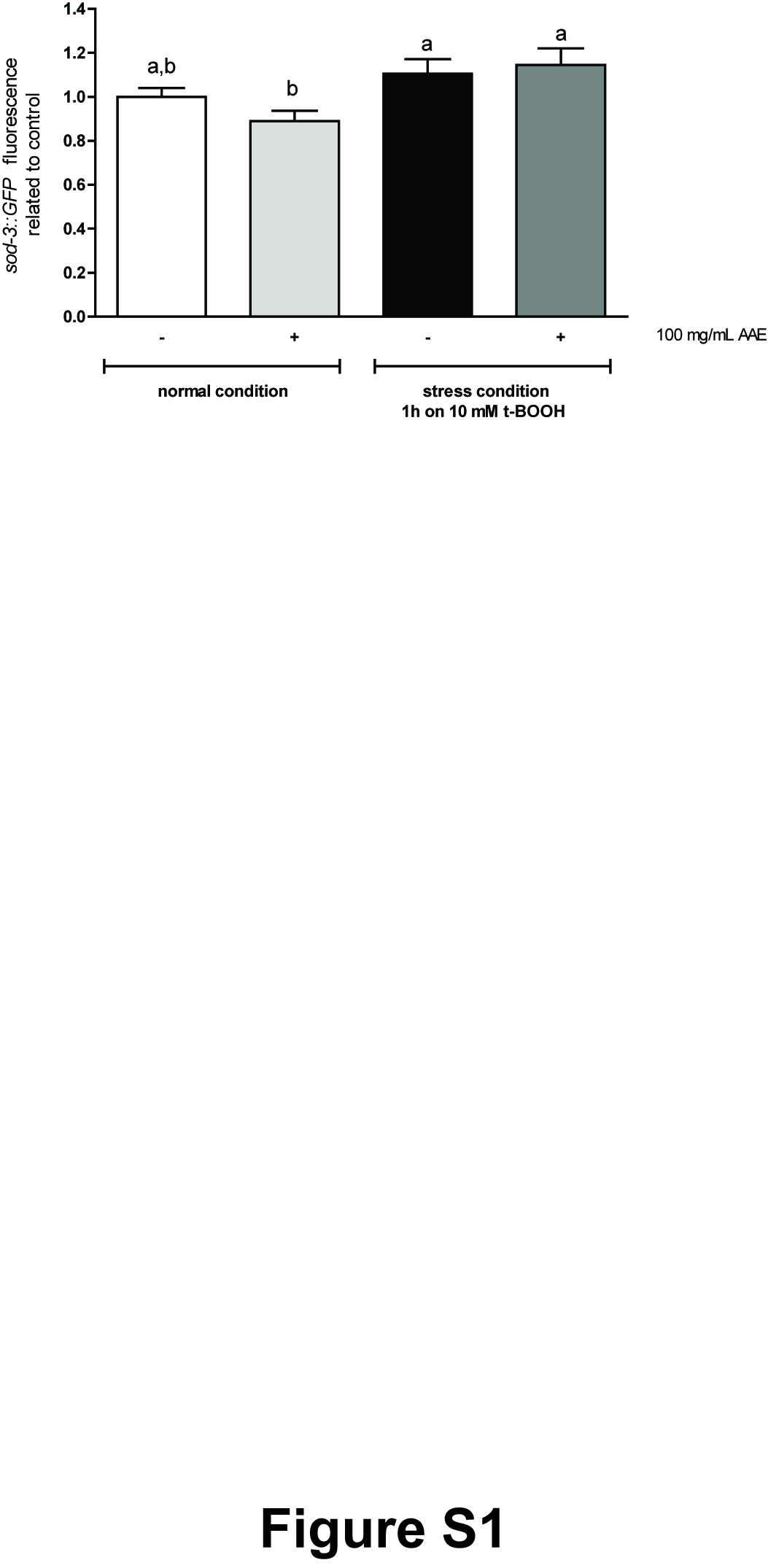

Supplement: Figure S1 — Effect of açaí aqueous extract (AAE) on sod-3::GFP expression. Transgenic worms carrying the reporter gene sod-3::GFP were treated with control solution (S basal) or 100 mg/mL AAE for 48 h from L1 and then with or without the oxidative stress condition. After a 1-h recovery period, photographs were taken on a fluorescence microscope. GFP fluorescence signals were measured using NIH Image J software. Different letters correspond to significant differences by the Kruskal-Wallis test followed by Dunn's post-test. AAE treatment alone nor the exposure to 10 mM t-BOOH for 1 h significantly upregulated sod-3::GFP expression. (TIF) [file pone.0089933.s001.tif]
