# Supplementary material for: Açaí (Euterpe oleracea Mart.) Modulates Oxidative Stress Resistance in Caenorhabditis elegans by Direct and Indirect Mechanisms
Source: PLoS One. 2014 Mar 3;9(3):e89933. doi: 10.1371/journal.pone.0089933 (PMC3940722; doi:10.1371/journal.pone.0089933)
Supplement: Table S1 — List of primers for qPCR. (DOCX) [file pone.0089933.s002.docx]

**Table S1.** List of primers for qPCR

| **Gene** | **Primers sequence**  **(5’ → 3’)** | **Reference** |
| --- | --- | --- |
| *daf-16* | F- TCAGGGATAAGGGAGATTCG  R- CAGATTGTGACGGATCGAGTT | Wilson et al., 2006 |
| *sod-3* | F- AGCATCATGCCACCTACGTGA  R- CACCACCATTGAATTTCAGCG | Sangha et al., 2012 |
| *ctl-1* | F- AATGGATACGGAGCGCATAC  R- TCCTGTTCAGCACCATCTTG | Vigneshkumar et al., 2011 |
| *gst-7* | F- GACAGCTTCCACTCCTTGAA  R- GCACACTTTCCATTGATTCC | Tullet et al., 2008 |
| *osr-1* | F- AGCATCATGCCACCTACGTGA  R- CACCACCATTGAATTTCAGCG | Xue et al., 2011 |
| *ama-1* | F-CCTACGATGTATCGAGGCAAA  R-CCTCCCTCCGGTGTAATAATG | Hoogewijs et al., 2008 |
